# Impact of role conflict and job stress on turnover intention among Korean physician assistant nurses: A cross-sectional study

**DOI:** 10.1016/j.ijnss.2026.02.014

**Published:** 2026-02-11

**Authors:** Jin-Won Lee, Eun-Hi Choi, Ji-Sun Back

**Affiliations:** a Department of Nursing, Daejeon Eulji Medical Center, Daejeon, the Republic of Korea; b Department of Nursing, Eulji University, Gyeonggi-do, the Republic of Korea; c Department of Nursing, Dongnam Health University, Gyeonggi-do, the Republic of Korea

**Keywords:** Job stress, Nursing legislation, Physician assistant nurses, Role conflict, Turnover intention

## Abstract

**Objectives:**

This study aimed to investigate the relationships among role conflict, job stress, and turnover intention among physician assistant (PA) nurses in the Republic of Korea and to identify key influencing factors of turnover intention.

**Methods:**

A cross-sectional descriptive study was conducted with 120 PA nurses recruited through a professional PA network in Gyeonggi Province and Daejeon Metropolitan City between June 1 and September 30, 2023. Validated instruments were used to measure role conflict, job stress, and turnover intention. Pearson correlation analysis was used to examine the associations between these variables, and multiple regression analysis was performed to identify influencing factors while controlling for demographic, organizational, and psychological covariates.

**Results:**

PA nurses reported relatively high levels of role conflict (3.66 ± 0.60), particularly in the subdomains of role ambiguity and work overload, moderate levels of job stress (2.78 ± 0.21) and higher levels of turnover intention (3.80 ± 0.64). Correlation analysis showed a strong positive association between role conflict and turnover intention (*r* = 0.604, *P* < 0.001), while job stress was moderately correlated with turnover intention (*r* = 0.236, *P* = 0.009). Notably, role conflict and job stress were not significantly associated with each other (*r* = 0.066, *P =*0.476). In multiple regression analysis, turnover intention was significantly associated with role conflict (*β* = 0.487, *P* < 0.001), educational level (*β* = 0.314 for master’s degree or higher; *β* = 0.288 for bachelor’s degree), and job stress (*β* = 0.171, *P* = 0.017). The final model explained 47.0 % of the variance in turnover intention (Adjusted *R*^2^ = 0.437, *F* = 19.488, *P* < 0.001).

**Conclusions:**

This study identified key factors influencing turnover intention, including role conflict (primarily driven by role ambiguity and work overload), educational level, and job stress. These findings suggest that establishing clear role definitions, enhancing legal recognition of PA nurses, providing ongoing professional education, and implementing stress management programs are essential strategies for improving PA nurse retention and healthcare workforce stability.

## What is known?

• Physician assistant (PA) nurses in the Republic of Korea work under ambiguous role boundaries and limited legal protection, which are associated with retention challenges.
• Previous studies have mainly focused on job satisfaction or retention intention, often examining role conflict and job stress as separate constructs rather than investigating their interconnected roles in turnover intention.

## What is new?

• This study identified strong positive relationships among role conflict, job stress, and turnover intention, with role conflict showing the strongest association. Notably, role conflict and job stress were not significantly correlated, suggesting distinct pathways to turnover intention.
• Multiple regression analysis revealed that role conflict (primarily driven by role ambiguity and work overload) is the strongest factor of turnover intention, followed by educational level and job stress. Higher educational attainment was associated with increased turnover intention, suggesting a mismatch between educational investment and professional recognition.
• These findings suggest that establishing clear role definitions in the Nursing Act, implementing formal written job descriptions, providing ongoing professional education aligned with career pathways, and developing stress management programs are essential strategies for improving PA nurse retention and healthcare workforce stability in the Republic of Korea.

## 1 Introduction

Physician assistants (PAs) have been adopted internationally as a strategy to address healthcare workforce shortages. While PA models vary across nations, many established PA systems—particularly in North America—are characterized by independent professional licensure and standardized training programs [1,2]. In contrast, the Korean PA nurse system differs fundamentally in that it is grounded in nursing licensure rather than a separate PA credential. It operates through institution-specific, physician-supervised delegated medical tasks without standardized national training or legal recognition [3].

In the Republic of Korea, advanced practice nurses (APNs) are registered nurses with specialized master’s-level certification in designated areas [4]; however, some APNs choose to work in PA nurse roles. “PA nurses” were defined as registered nurses currently performing physician-assistant-related duties under physician supervision in hospital settings, regardless of their formal certification as APNs [5,6]. Korean PA nurses are often selected to fill roles vacated by residents in specialties that physicians tend to avoid, such as thoracic, general, obstetric-gynecologic, and neurosurgery. These individuals are typically experienced nurses with long tenures in their departments [5]. The demand for PA nurses in the Republic of Korea increased following the enactment of the Resident Special Act in 2017, which restricted resident working hours. The number of PA nurses rose from 1,009 in 2010 to 5,619 in 2022, a five-fold increase reflecting consistent annual growth [7]. Although these professionals are generally composed of skilled, experienced nurses, they have historically worked autonomously without explicit legal protections, standardized educational training, or a formally defined scope of practice [5,8]. As gaps in healthcare delivery widened due to tensions surrounding medical school enrollment expansion policies in 2024, PA nurses emerged as essential contributors to the Korean healthcare workforce. Policy discourse is increasingly centered on formal legislation to recognize and regulate PA nurse roles [9].

However, the current responsibilities of Korean PA nurses are shaped by institutional convenience and delegated physician tasks, leading to unclear role boundaries and a lack of specialized or defined duties [6,10]. These conditions generate substantial role conflict and interpersonal strain, well-documented occupational stressors in nursing [11]. From an occupational and organizational perspective, unclear role boundaries and conflicting expectations contribute to work strain and psychological distress [12]. Qualitative studies of PA nurses’ work experiences have documented multiple manifestations of such role conflict, including identity confusion arising from an ambiguous position between physicians and nurses [13,14]. PA nurses report challenges such as undefined scopes of practice, insufficient formal education systems, dual supervision structures, conflicts with nursing departments, and uncertainty about their professional future, indicating that role conflict is a pervasive feature of their work environment [15,16].

Role conflict serves as a significant source of job stress among PA nurses [17]. Although PA nurses possess the professional competencies to perform tasks associated with both nursing and physician roles, they experience anxiety and lowered self-esteem due to having to work under physician supervision and document their clinical contributions under physicians’ names rather than their own [18]. Role conflict—often arising from role ambiguity, work overload, and inadequate interprofessional coordination—increases psychological burden and contributes to elevated job stress [19,20]. PA nurses face significant workplace challenges, including unclear role definitions and a lack of professional recognition, which contribute to role conflict and job stress [14,17]. High job stress is, in turn, associated with increased turnover intention, as individuals attempt to cope with chronic strain by withdrawing from the role or the organization [13,[21], [22], [23]]. Previous Korean studies have reported that turnover intention among PA nurses is comparable to or slightly higher than that observed among general hospital nurses [24,25]. The interrelationships among these three factors suggest a potential cascade mechanism, in which psychological stressors may sequentially influence turnover intention [26]. This is particularly concerning because the loss of skilled PA nurses negatively affects hospital management efficiency and undermines patient care quality, emphasizing the urgent need to prevent PA nurse attrition [27].

Although legal protections for PA nurses were clarified through the enactment of the Nursing Act in August 2024, substantial societal debate remains regarding the delineation of their specific duties [28]. Korean PA nurses may exhibit distinct patterns due to the institutional framework rooted in nursing licensure and historically limited legal and educational standardization [13]. Previous studies [[14], [22], [29], [30]] on Korean PA nurses have primarily focused on factors influencing job satisfaction or, in limited contexts, retention intention. However, the relationships among these three factors have not been systematically examined among Korean PA nurses.

Therefore, this study aims to investigate the relationships among role conflict, job stress, and turnover intention in PA nurses in the Republic of Korea, and to identify key influencing factors of turnover intention while controlling for relevant demographic, organizational, and psychological factors. By clarifying these relationships and identifying modifiable factors, this study seeks to provide evidence-based insights to develop targeted interventions that improve PA nurse retention and strengthen the PA nurse workforce.

## 2 Methods

### 2.1 Study design

This study employed a descriptive, cross-sectional survey design and was reported in accordance with the Strengthening the Reporting of Observational Studies in Epidemiology (STROBE) guidelines for cross-sectional studies [31].

### 2.2 Study participants

Participants were recruited through a professional PA nurse network between June 1 and September 30, 2023. Participants were employed in hospitals located in Gyeonggi Province and Daejeon Metropolitan City. To protect participant anonymity and confidentiality, information on hospital names and the number of hospitals involved was not collected, as such details could potentially enable identification of individuals within the PA network.

The inclusion criteria were: 1) nurses performing PA nurse duties, 2) nurses currently engaged in medical tasks under physician supervision. The exclusion criteria were those who held legally recognized qualifications in any of the 13 nursing specialties and did not perform physician-assigned tasks. However, APNs who were carrying out PA nurse responsibilities were included. In the Republic of Korea, APNs are registered nurses who complete specialized master’s-level education programs in one of 13 specialty areas designated under the Korean Medical Service Act and obtain official certification from the Ministry of Health and Welfare [4]. These legally recognized APNs are distinguished from PA nurses, who operate without a specific legal framework or standardized certification. Despite holding APN credentials, some nurses choose to work in PA nurse positions performing physician-delegated tasks. Therefore, individuals with APN qualifications who were actively engaged in PA nurse duties were included in this study, as their work experience reflected the PA nurse role.

The required sample size was calculated using G∗Power 3.1.9.7, assuming an effect size of 0.15, a significance level of 0.05, and a statistical power of 0.80 for multiple regression analysis. The minimum sample size was determined to be 109. Considering a 10 % dropout rate, the target sample was set at 120.

### 2.3 Measures

Individual factors (gender, age, educational level, and work experience) were included as these demographic characteristics have been consistently associated with turnover intention in nursing populations [23]. Organizational factors, including the work instruction department, the clinical department, written job regulations, and the availability of regular job-related education, were selected because institutional structures and educational support are known to influence role clarity and job satisfaction. Psychological factors—selection motivation for the PA nurse role, clinical department satisfaction, and anxiety due to lack of legal protection—were included to capture subjective perceptions specific to the PA nurse context, as these attitudinal variables may independently affect retention beyond objective work conditions.

#### 2.3.1 Individual personal factors

Individual personal factors were defined as demographic and career-related characteristics, including gender, age, educational level, and work experience.

#### 2.3.2 Organizational factors

Organizational factors were defined as workplace-related conditions and institutional structures that shape PA nurses’ work environments. These included the work instruction department, clinical department, written job regulations for PA nurses, and regular job-related education programs.

#### 2.3.3 Psychological factors

Psychological factors included selection motivation for PA nurses, current clinical department satisfaction, and anxiety due to a lack of legal protection.

#### 2.3.4 Role conflict

Role conflict was measured using Kim and Park’s Role Conflict Scale for clinical nurses [32], which was later modified for APNs by Lee et al. [33]. The instrument includes 34 items across four subdomains: role ambiguity (15 items), lack of ability (10 items), work overload (4 items), and lack of cooperation (5 items). Each item was rated on a 5-point Likert scale ranging from 1 (strongly disagree) to 5 (strongly agree), with higher scores indicating greater perceived role conflict. The reliability of the instrument in Lee et al.’s [33] study was 0.94 (Cronbach’s α coefficient), with subdomain reliabilities of 0.91 for role ambiguity, 0.89 for lack of ability, 0.87 for work overload, and 0.70 for lack of cooperation. In this study, the overall reliability was 0.96 (Cronbach’s α coefficient), with subdomain reliabilities of 0.92 for role ambiguity, 0.92 for lack of ability, 0.80 for work overload, and 0.81 for lack of cooperation.

#### 2.3.5 Job stress

Job stress was measured using Park’s Job Stress Instrument [34], which was adapted by Lee [35] for nurses working in PA roles and designed to reflect the organizational characteristics of hospital settings. The instrument includes 18 items across three subdomains: job role factors (14 items), structural factors (2 items), and interpersonal relationship factors (2 items). Each item was rated on a 5-point Likert scale ranging from 1 (strongly disagree) to 5 (strongly agree), with higher scores indicating greater perceived job stress. The reliability of Park’s original instrument was 0.87 (Cronbach’s α coefficient). In the present study, the overall reliability was 0.75 (Cronbach’s α coefficient). Subdomain reliabilities were 0.72 for job role factors, 0.57 for structural factors, and 0.84 for interpersonal relationship factors. The relatively low reliability for structural factors (0.57) may be attributable to the subdomain containing only two items. Although the reliability of the structural factors subscale was relatively low, this subdomain consists of only two items, and Cronbach’s α coefficient is known to be sensitive to the number of items in a scale [36]. Structural factors were retained because they capture organizational-level stressors that may vary substantially across hospital settings rather than reflecting individual-level consistency.

#### 2.3.6 Turnover intention

Turnover intention was measured using an instrument developed initially by Mobley et al. [37], modified by Lee [38] for office workers, and further adapted by Ham [39] for nurses. The instrument consists of 13 items assessing thoughts about leaving, job search intentions, and plans to quit one’s current organization (e.g., “I want to work at a different hospital,” “I often think about quitting the hospital,” “I am planning to search for another hospital actively”), each rated on a 5-point Likert scale ranging from 1 (strongly disagree) to 5 (strongly agree), with higher scores reflecting greater turnover intention. The reliability reported in Ham’s [39] study was 0.89 (Cronbach’s α coefficient), while the reliability in the present study was 0.92 (Cronbach’s α coefficient).

### 2.4 Data collection

The online survey was developed using a secure web-based platform and distributed via the social media networks of PA nurses in these regions. All data were collected anonymously, and participants provided informed consent after being informed of the study purpose and procedures. Participation was voluntary, and participants were informed of their right to withdraw at any time without penalty. Data were collected using a self-administered online questionnaire. The survey took approximately 15–20 min to complete. All responses were submitted anonymously through the online platform. Because the online survey system was designed to prevent submission of incomplete responses, 120 fully completed questionnaires were collected, yielding a response rate of 100 %.

### 2.5 Data analysis

Data were analyzed using IBM SPSS Statistics for Windows version 27.0. Descriptive statistics were calculated for all variables; categorical variables were summarized using frequencies and percentages, while continuous variables were expressed as means and standard deviations. Differences in study variables by participant characteristics were examined using an independent-samples *t*-test for two-group comparisons and a one-way analysis of variance (ANOVA) for comparisons across three or more groups. Scheffe’s test was applied for post hoc pairwise comparisons when ANOVA indicated significant group differences. Relationships among the core variables were analyzed using Pearson’s correlation coefficients.

Multiple regression analysis was performed to identify influencing factors of turnover intention. Considering theoretical relevance, empirical patterns, and the need to ensure model stability given the sample size, a parsimonious multivariable regression model was constructed [40]. Before regression analysis, assumptions of linearity, independence of errors, homoscedasticity, and normality of residuals were examined. Multicollinearity was assessed using variance inflation factors (VIFs; acceptable if < 10) and tolerance values (acceptable if > 0.1). Variables were entered into the regression model in a hierarchical manner: demographic and organizational factors were entered first, followed by psychological variables, role conflict, and job stress. Statistical significance was set at *P* < 0.05.

### 2.6 Ethical considerations

This study received approval from the Institutional Review Board (IRB) of Eulji University (IRB No. EU23-47) before data collection. The study purpose was explained to leaders of PA associations in the two target provinces, and their permission was obtained.

## 3 Results

### 3.1 General characteristics and differences in turnover intention

Slightly more than half of the participants were female (54.2 %), and nearly half were under 30 years of age (47.5 %). The mean age was 30.78 ± 4.35 years. Most participants held a bachelor’s degree (80.8 %), and 36.7 % had less than 2 years of experience as PA nurses. Regarding organizational characteristics, 45.0 % reported receiving work instructions primarily from medical departments, while 43.3 % received instructions from both medical and nursing departments. Most participants reported the absence of written job regulations (90.0 %) and regular job-related education programs (95.0 %). The most common reasons for selecting the PA nurse role were avoiding three-shift work (39.2 %), followed by involuntary assignment (29.2 %) and pursuit of professional or specialized work (23.3 %). The detailed characteristics of the participants are shown in Appendix A.

### 3.2 Correlations among role conflict, job stress, and turnover intention

The mean role conflict score was 3.66 ± 0.60 on a 5-point scale. Among subdomains, role ambiguity (3.84 ± 0.63) and work overload (3.84 ± 0.71) showed the highest scores, followed by lack of cooperation (3.67 ± 0.71) and lack of ability (3.33 ± 0.65). The mean job stress score was 2.78 ± 0.21. Structural factors showed the highest subdomain score (3.31 ± 0.60), followed by job role factors (2.79 ± 0.24) and interpersonal relationship factors (2.18 ± 0.42). The mean turnover intention score was 3.80 ± 0.64, indicating a relatively high level of turnover intention among participants.

Pearson’s correlation analysis showed that role conflict was strongly positively correlated with turnover intention (*r* = 0.604, *P* < 0.001). Job stress was also positively correlated with turnover intention (*r* = 0.236, *P* = 0.009). However, role conflict and job stress showed no significant correlation (*r* = 0.066, *P* = 0.476).

### 3.3 Factors influencing turnover intention

In the single-factor analysis, participants with a bachelor’s or master’s degree reported higher turnover intention than those with a college degree (*F* = 12.616, *P* < 0.001). Higher turnover intention was also observed among participants receiving work instructions from medical departments (*F* = 7.451, *P* < 0.001) and among those without regular job-related education programs (*t* = −2.499, *P* = 0.014).

Multiple regression analysis identified role conflict (*β* = 0.487, *P* < 0.001), educational level (*β* = 0.314 for master’s degree or higher; *β* = 0.288 for bachelor’s degree), and job stress (*β* = 0.171, *P* = 0.017) as significant influencing factors of turnover intention (Table 1). The final model explained 47.0 % of the variance in turnover intention (Adjusted *R*^*2*^ = 0.437, *F* = 19.488, *P* < 0.001). Multicollinearity was not detected (VIFs range: 1.048–3.637; tolerance range: 0.275–0.954; Durbin–Watson = 2.007).

**Table 1:** Factors influencing turnover intention among physician assistant nurses (*n* = 120).

Variables	*B*	*β*	*t*	*P*	95 %*CI*
Constant	−0.308	–	−0.490	0.623	–
Educationaal level (ref: College)					
Bachelor’s degree	0.469	0.288	2.888	0.005	0.151, 0.787
Master’s degree or above	0.697	0.314	3.348	<0.001	0.289, 1.105
Work instruction department (ref: Nursing department)					
Medical department	0.058	0.045	0.346	0.731	−0.271, 0.387
Both medical and nursing departments	0.053	0.041	0.338	0.736	−0.254, 0.360
Regular job-related education programs (ref: Yes)					
No	0.338	0.115	1.481	0.140	−0.109, 0.785
Role conflict	0.520	0.487	5.654	<0.001	0.340, 0.700
Job stress	0.500	0.171	2.427	0.017	0.096, 0.904

*Note: R*^2^ = 0.470, Adjusted *R*^2^ = 0.437, *F* = 19.488, *P* < 0.001, Durbin-Watson = 2.007.

## 4 Discussion

### 4.1 Relationships among role conflict, job stress, and turnover intention

This study found strong positive relationships among role conflict, job stress, and turnover intention in Korean PA nurses, with role conflict showing the strongest association. Role conflict and job stress were not significantly correlated, suggesting distinct pathways to turnover intention. Role ambiguity, which showed the highest score among role conflict subdomains, has been consistently reported as a key source of dissatisfaction in international PA literature. Studies from Germany and the United Kingdom similarly highlight unclear role authority and the absence of formally defined PA roles as persistent challenges within healthcare systems [41,42]. In the Republic of Korea, PA nurses are positioned between physicians and nurses, often operating within blurred professional boundaries that contribute to role confusion and identity strain [29]. In the present study, only a small proportion of participants reported having written job descriptions, in contrast to countries such as the United States and Canada, where PA roles are supported by legally established scopes of practice and standardized job descriptions [43,44]. The absence of institutionalized role definitions in the Republic of Korea means that PA nurses frequently perform extended medical duties without clear legal safeguards, a condition that may exacerbate anxiety and moral discomfort [45] and intensify role conflict.

Structural factors—such as promotion systems, compensation, and working conditions—emerged as the most prominent dimension of job stress, consistent with prior research [21]. Similar structural stressors have been identified as significant sources of dissatisfaction among American PAs, suggesting that these challenges reflect a broader, cross-national pattern within the PA profession [46,47]. PA nurses often derive professional satisfaction from clinical autonomy and expertise developed through extensive nursing experience [48], attributes that are also strongly associated with job satisfaction among American PAs [46,47]. However, despite these professional strengths and clinical competencies, Korean PA nurses frequently face institutional barriers in which their contributions are recorded under physicians’ names and their individual performance is challenging to evaluate. This disconnect between professional expertise and formal institutional recognition may intensify job stress, particularly among more experienced and educated nurses.

### 4.2 Factors influencing turnover intention

Multiple regression analysis identified role conflict, educational level, and job stress as significant influencing factors of turnover intention. Role conflict was the most salient psychological factor associated with turnover intention among PA nurses. These findings highlight the importance of explicitly defining PA nurses’ roles in the Nursing Act and of implementing formal, written job descriptions at the institutional level.

Among individual personal factors examined, only educational attainment showed a significant association with turnover intention. Higher levels of education were associated with greater turnover intention, a finding consistent with previous research [24] and with studies reporting lower job satisfaction among more highly educated PA nurses [21]. This association suggests a mismatch between educational investment and the professional experiences of PA nurses. Advanced education may heighten expectations regarding professional recognition, role utilization, and career development. When such expectations are unmet, particularly in roles with limited formal educational differentiation, frustration and disengagement may increase. Although continuing professional development is generally linked to positive behavioral change among healthcare professionals [49], its benefits may be diminished when educational advancement is not meaningfully reflected in professional roles.

In the Korean healthcare context, PA nurses play a critical role in addressing physician shortages and maintaining service continuity, a trend also observed internationally among Ps and APNs [[50], [51], [52]]. However, in the absence of clearly articulated career pathways, educational differentiation, and transparent evaluation systems, highly educated PA nurses may be more likely to consider leaving their positions. These findings suggest the need to align PA nurses’ educational attainment with clear and differentiated career pathways, potentially through linkage with recognized advanced practice nursing roles.

Job stress also contributed to turnover intention, although its influence was weaker than that of role conflict. In the Korean context, these professional strengths may coexist with heightened stress when institutional structures fail to provide clear evaluation criteria and career trajectories. International models, particularly those in the United States and Canada, suggest that structured certification systems, defined departmental affiliations, and transparent promotion pathways can mitigate job stress and support workforce retention [47,53]. Adapting such frameworks to the Korean PA nurse system may therefore be critical for reducing psychological strain and enhancing long-term retention.

Regarding organizational factors, several variables—including the presence of written job regulations and the availability of regular job-related education programs—showed significant associations with turnover intention in univariate analyses. In particular, PA nurses who had access to regular education programs reported significantly lower turnover intention. These findings are consistent with previous studies demonstrating higher job satisfaction among PA nurses who received pre-deployment or ongoing education [17,21,54], as well as international evidence emphasizing the role of structured onboarding and continuous professional development in reducing turnover among PAs [55,56].

However, these organizational factors did not remain significant influencing factors in the final multivariable regression model. This pattern suggests that their influence on turnover intention may operate indirectly through psychological mechanisms, particularly role conflict and job stress, rather than exerting a direct effect. In other words, organizational conditions may constitute foundational factors that shape the psychological work environment, but their effects become less visible once proximal psychological variables are taken into account. Qualitative evidence supports this interpretation. Korean PA nurses often begin clinical duties without systematic preparatory education and are required to learn tasks independently because formal training structures are absent [18]. Such conditions may not immediately manifest as turnover intention but can contribute to sustained role ambiguity and job stress. This contrasts sharply with Canada's formalized PA education model, which emphasizes comprehensive competency development based on the Entrustable Professional Activities for PA (EPA-PA) and Canadian Medical Education Directives for Specialists PA (CanMEDS-PA) frameworks [53]. Establishing systematic education programs and clear institutional structures for PA nurses would address these foundational gaps, mitigating the psychological strain that ultimately drives turnover intention.

### 4.3 Limitations

This study has several limitations. First, participants were recruited through convenience sampling from a PA nurse network in two regions (Gyeonggi Province and Daejeon Metropolitan City), which may limit generalizability. In addition, information on hospital names and the number of participating institutions was not collected to ensure anonymity, restricting detailed institutional-level analysis. Future studies using nationally representative samples are warranted, along with field-specific analyses to capture variations across clinical specialties. Second, this study focused on PA nurses employed before the 2024 healthcare crisis and may not reflect the experiences of newly deployed PA nurses after the crisis. Longitudinal research is needed to examine adaptation and turnover patterns in this emerging workforce. Third, although some participants were certified APNs, all were included based on their current PA duties; thus, role heterogeneity cannot be entirely excluded and should be examined in future studies. Fourth, the reliability of the structural factors subdomain of the job stress scale was relatively low, which may affect the precision of job stress measurement in this study. Although this subdomain consists of only two items and Cronbach’s α coefficient is known to be sensitive to the number of items in a scale, this limitation should be considered when interpreting findings related to job stress. Finally, because this is a cross-sectional study, causal relationships cannot be established. The finding that organizational factors were significant in univariate analyses but not in the multivariable model suggests the presence of potential indirect effects via psychological mechanisms. Future research employing hierarchical regression, mediation analysis, or structural equation modeling would help clarify these pathways.

Despite these limitations, this study is among the first empirical investigations of turnover intention among PA nurses in the Republic of Korea. Conducted at a pivotal moment marked by the enactment of the Nursing Act and the institutionalization of the PA nurse system, it provides timely evidence to inform policy and organizational strategies to support the integration and long-term retention of PA nurses in Korea’s healthcare system.

## 5 Conclusions

This study examined the strong positive relationships among the three variables, with role conflict showing the strongest association with turnover intention, followed by job stress. Notably, role conflict and job stress were not significantly correlated, suggesting distinct pathways to turnover intention. Role conflict was the strongest factor of turnover intention, followed by educational level and job stress. Role conflict, primarily driven by role ambiguity and work overload, emerged as the most salient psychological factor. Higher educational attainment was associated with increased turnover intention, suggesting a mismatch between educational investment and professional recognition. Organizational factors, particularly the availability of regular education programs, showed significant associations in univariate analyses, suggesting that their influence may operate indirectly through psychological mechanisms. These findings underscore the importance of establishing clear role definitions, enhancing legal recognition of PA nurses, providing ongoing professional education, and implementing stress management programs as essential strategies for improving PA nurse retention and healthcare workforce stability.

## Data availability statement

The datasets generated during and/or analyzed during the current study are available from the corresponding author upon reasonable request.

## CRediT authorship contribution statement

**Jin-Won Lee:** Project administration, Data curation, Validation, Formal analysis, Writing - original draft. **Eun-Hi Choi:** Supervision, Conceptualization, Methodology, Software, Project administration, Writing - original draft, Writing - review & editing, Visualization. **Ji-Sun Back:** Conceptualization, Resources, Writing - original draft, Writing - review & editing.

## Declaration of competing interest

The authors have declared no conflict of interest.
