# Supplementary material for: Impact of role conflict and job stress on turnover intention among Korean physician assistant nurses: A cross-sectional study
Source: Int J Nurs Sci. 2026 Feb 11;13(2):172–8. doi: 10.1016/j.ijnss.2026.02.014 (PMC13044359; doi:10.1016/j.ijnss.2026.02.014)
Supplement: Multimedia component 1 [file mmc1.docx]

**角色冲突与工作压力对韩国助理护士离职意向的影响：一项横断面研究**

Jin-Won Lee, Eun-Hi Choi, Ji-Sun Back

【**摘要**】
**目的** 探讨韩国助理护士角色冲突、工作压力与离职意向之间的关系，并分析离职意向的影响因素。

**方法** 采用横断面研究设计。2023年6月1日至9月30日，通过专业助理护士网络选取韩国京畿道和大田市120名助理护士为调查对象。采用经过验证的角色冲突、工作压力及离职意愿量表收集相关数据。采用Pearson相关分析变量间关联；采用多元回归分析在控制人口学、组织及心理学协变量后，识别离职意向的影响因素。

**结果** 助理护士的角色冲突平均得分较高（3.66±0.60），其中以角色模糊与工作负荷维度突出；工作压力处于中等水平（2.78±0.21）；离职意向为（3.80±0.64）分。相关性分析结果显示，角色冲突与离职意向呈强正相关（*r*=0.604，*P*<0.001）；工作压力与离职意愿呈中度正相关（*r*=0.236，*P*=0.009）；而角色冲突与工作压力之间无显著相关性（*r*=0.066, *P*=0.476)）。多元回归分析结果表明，离职意向与角色冲突（*β*=0.487，*P*<0.001）、教育水平（硕士及以上学历：*β*=0.314；本科学历：*β*=0.288）及工作压力（*β*=0.171，*P*=0.017）显著相关，模型共解释离职意愿47.0%的变异量（调整后*R*²=0.437，*F*=19.488，*P*<0.001）。

**结论** 角色冲突、教育水平及工作压力是韩国助理护士离职意向的重要影响因素。未来应着力明确助理护士的角色定位、推动法律层面的角色认可、提供持续性专业教育，并制订系统的压力管理方案，以提升其留职意向，保障护士队伍的稳定性。

【**关键词**】工作压力；护理法规；助理护士；角色冲突；离职意向

**通信作者：**Eun-Hi Choi，E-mail：[choieh@eulji.ac.kr](mailto:choieh@eulji.ac.kr)
