# Supplementary material for: Impact of role conflict and job stress on turnover intention among Korean physician assistant nurses: A cross-sectional study
Source: Int J Nurs Sci. 2026 Feb 11;13(2):172–8. doi: 10.1016/j.ijnss.2026.02.014 (PMC13044359; doi:10.1016/j.ijnss.2026.02.014)
Supplement: Multimedia component 2 [file mmc2.docx]

Appendix A General characteristics of the participants and differences in turnover intention (*n* = 120).

| Characteristics | | *n* (%) | Turnover intention | | |
| --- | --- | --- | --- | --- | --- |
|  |  |  | *Mean* ± *SD* | *t*/*F* | *P* (Scheffe) |
| Individual personal factors | | | | | |
| Gender | Male | 55 (45.8) | 3.84 ± 0.57 | 0.620^a^ | 0.537 |
|  | Female | 65 (54.2) | 3.77 ± 0.69 |  |  |
| Age (years) | <30 | 57 (47.5) | 3.78 ± 0.68 | 0.292^b^ | 0.747 |
|  | 30–34 | 43 (35.8) | 3.86 ± 0.68 |  |  |
|  | ≥35 | 20 (16.7) | 3.72 ± 0.42 |  |  |
| Educational level | College ^c^ | 12 (10.0) | 3.01 ± 0.99 | 12.616^b^ | <0.001  (c<d,e) |
|  | Bachelor’s degree ^d^ | 97 (80.8) | 3.87 ± 0.53 |  |  |
|  | Master’ degree or above^e^ | 11 (9.2) | 4.06 ± 0.41 |  |  |
| Work experience  (years) | <2 | 44 (36.7) | 3.86 ± 0.68 | 0.825^b^ | 0.483 |
|  | 2–<3 | 20 (16.7) | 3.90 ± 0.53 |  |  |
|  | 3–<5 | 34 (28.3) | 3.78 ± 0.73 |  |  |
|  | ≥5 | 22 (18.3) | 3.62 ± 0.47 |  |  |
| Organizational factors | | | | | |
| Work instruction  department | Nursing department ^f^ | 14 (11.7) | 3.50 ± 0.61 | 7.451^b^ | <0.001  (f,h<g) |
|  | Medical department ^g^ | 54 (45.0) | 4.03 ± 0.41 |  |  |
|  | Both medical and nursing departments ^h^ | 52 (43.3) | 3.64 ± 0.75 |  |  |
| Clinical department | Internal Medicine | 23 (19.2) | 3.82 ± 0.55 | 0.525^b^ | 0.788 |
|  | General Surgery | 30 (25.0) | 3.74 ± 0.75 |  |  |
|  | Orthopedics Surgery | 19 (15.8) | 3.99 ± 0.57 |  |  |
|  | Neuro Surgery | 9 (7.5) | 3.61 ± 1.03 |  |  |
|  | Cardiothoracic Surgery | 11 (9.2) | 3.77 ± 0.70 |  |  |
|  | Obstetrics Gynecology | 16 (13.3) | 3.86 ± 0.43 |  |  |
|  | Other^*^ | 12 (10.0) | 3.69 ± 0.45 |  |  |
| Written job regulations | Yes | 12 (10.0) | 3.50 ± 0.70 | -1.711^a^ | 0.090 |
|  | No | 108 (90.0) | 3.83 ± 0.63 |  |  |
| Regular job-related  education programs | Yes | 6 (5.0) | 3.17 ± 0.52 | -2.499^a^ | 0.014 |
|  | No | 114 (95.0) | 3.83 ± 0.63 |  |  |
| Psychological factors | | | | | |
| Selection motivation | Involuntary assignment | 35 (29.2) | 3.87 ± 0.60 | 0.493^b^ | 0.741 |
|  | Avoid three-shift work | 47 (39.2) | 3.83 ± 0.63 |  |  |
|  | Professional work | 28 (23.3) | 3.74 ± 0.75 |  |  |
|  | Self-improvement | 7 (5.8) | 3.71 ± 0.48 |  |  |
|  | Other (reinstatement) | 3 (2.5) | 3.41 ± 0.55 |  |  |
| Clinical department satisfaction | Satisfaction | 111 (92.5) | 3.81 ± 0.65 | 0.297^a^ | 0.767 |
|  | Dissatisfaction | 9 (7.5) | 3.74 ± 0.55 |  |  |
| Anxiety due to lack  of legal protection | Yes | 27 (22.5) | 3.90 ± 0.58 | 0.990 | 0.323 |
|  | No | 93 (77.5) | 3.77 ± 0.66 |  |  |

*Note:* ^*^ Neurology, Pediatrics, Emergency Medicine, Urology, Trauma, Ophthalmology, Hemodialysis. ^a^ independent sample *t*-test. ^b^ one-way analysis of variance.
